# Leveraging Innovative Financing Strategy to Increase Coverage and Resources Among Informal Sector for Social Health Insurance Within the Nigerian Context of Devolution: Evidence From Adoption Model Implementation

**DOI:** 10.3389/fpubh.2022.894330

**Published:** 2022-07-14

**Authors:** Simeon Beluonwu Onyemaechi, Uchenna Rita Ezenwaka

**Affiliations:** ^1^Anambra State Health Insurance Agency (ASHIA), Awka, Nigeria; ^2^Department of Health Administration and Management, Faculty of Health Science and Technology, College of Medicine, University of Nigeria Enugu, Enugu, Nigeria; ^3^Department of Pharmacology and Therapeutics, Health Policy Research Group, College of Medicine, University of Nigeria Enugu, Enugu, Nigeria

**Keywords:** adoption model, ASHIA, coverage, informal sector, innovative financing, resource mobilization, social health insurance scheme

## Abstract

**Background:**

Enrollment in sub-national social health insurance schemes (SSHIS) can be challenging in developing countries like Nigeria, particularly among people in the informal sector. This could be due to a lack of knowledge on its mode of operation and benefits, distrust in government, inimical religious and traditional beliefs, as well as constraining economic factors. A complementary and innovative financing strategy such as the philanthropist adoption model (ADM) could be beneficial in improving SSHIS coverage and financial resources among persons in the informal sector. The study provides new evidence on how ADM influenced health insurance coverage and resources within SSHIS among informal settings in Nigeria. It also highlights contextual factors influencing the implementation of ADM.

**Methods:**

This study employed a mixed-methods case-study approach undertaken in Anambra State, Southeast Nigeria. Data were collected through in-depth interviews (*n* = 14), document review (*n* = 12), and quantitative (enrollment data) methods. The respondents were purposively selected based on their involvement with the implementation of the SSHIS. Data analysis for qualitative data was done using the manual thematic framework approach while descriptive analysis was performed for the quantitative data.

**Results:**

The implementation of ADM was a valuable and effective strategy for improving knowledge, coverage, and resource mobilization (annual premium pool) within the SSHIS in the informal sector. The main enablers of the implementation of ADM include strong political will and commitment, wider stakeholders' consultation and collaboration, numerous public-spirited philanthropists, and legal institutionalization of health insurance. Other enablers include organizational factors like good teamwork among Anambra State Health Insurance Agency (ASHIA) staff, enabling work environment (incentives, supervision, office space), and experienced marketers in the agency. However, ADM had several barriers that affected its implementation—acceptability issues due to distrust for government and the health system, health systems barriers including substandard health facilities and equipment, and inimical cultural and religious beliefs about health insurance.

**Conclusion:**

The study demonstrates a case for the implementation of innovative ADM as a strategy for enhancing SSHIS financial sustainability and coverage of persons in the informal sector. Hence, the strategy should be adopted in settings where philanthropy abounds for increasing access to quality healthcare delivery to poor beneficiaries toward achieving universal health coverage in developing countries.

## Introduction

The desire to achieve Universal Health Coverage (UHC) has driven countries to seek and adopt suitable strategies of health care financing to ensure sustainable and universal access to quality, efficient and equitable healthcare for their citizens (1, 2). Health insurance as a financing mechanism provides the basis for achieving UHC goals due to its unique feature of pooling to ensure financial risk protection (3). For instance, an African country like Rwanda has been exemplified for advancing UHC through health insurance financing mechanisms with 78.7% population coverage (4). Unlike in Nigeria, the National Health Insurance Scheme (NHIS) has been struggling over the years with only about 4% population coverage of the formal sector since its introduction in 2005 (4, 5), hence minimal progress toward UHC. Consequently, the decentralization of the NHIS whereby the sub-national governments established their respective state social health insurance schemes (SSHIS) to extend health insurance coverage to a significant share of the population, address the huge health inequities, and high out-of-pocket expenditures (OOPE) toward achieving UHC (5, 6). However, the problem of operational sustainability of the health insurance policy is Nigeria's major challenge and by extension low- and middle-income countries (LMICs) (7).

Evidence shows that poor enrollment of persons in the informal sector into the SSHIS has been one of the major challenges of health insurance in Nigeria (8, 9) due to several challenges ranging from poor public awareness of health insurance policy and its benefits (9, 10), wrong perception or citizens lack of trust in government role in healthcare services, cultural and religious beliefs about health insurance attracting illness, amongst others. Hence, the poor coverage of informal sector enrollment in health insurance becomes worrisome given that Nigeria has a huge population within the informal setting (10).

A feasible and innovative healthcare financing strategy to improve coverage of the informal sector and increase resources within the SSHIS is through the philanthropists' “*Adoption Model”* (ADM). The ADM is designed to target a pool of public-spirited high-net-worth indigenes to pay premiums for low-income and vulnerable citizens thereby increasing the number of the informal sector and rural dwellers within the scheme. In this case, the public-spirited philanthropists are the “*adopters”*, and those who they pay premiums on their behalf become the “*adoptees*. The model leverages the existing traditional social support system to expand the informal sector pool in the SSHIS. The entire essence is to ensure that the adoptees” overtime benefits from health insurance then they would be well able to compare their pre- and post-health insurance expenditure on health care and possibly renew their health insurance policy regularly toward building a health insurance culture in the state. According to the Global Funds report, philanthropic investment funds allow diverse investors and groups to pool their financial contributions toward a cause. The pooled fund is then used to leverage financial resources to support health care programs in a more coordinated and efficient way, thereby improving access and quality of care (11).

The adoption of innovation could present an opportunity for change in an organization in response to healthcare demands. It is often postulated that the primary stimulus for an innovation adoption for organizational change emanates from the external environment, and hence influences organizational ability to innovate (11). Adoption usually starts with the recognition that a need exists and moves to search for solutions, then to the initial decision to attempt the adoption of a solution, and finally, to the actual decision to proceed with the implementation of the solution (12, 13). There is limited empirical evidence on the implementation of ADM in health insurance. However, existing literature shows that an end-to-end (E2E) innovation adoption model for the telemedicine process had been implemented in COVID-19 pandemic control in Brazil (14). Evidence indicated that the E2E model yielded a faster and more efficient outcome and, consequently, an optimization of the adoption process of telemedicine innovation (14).

In Nigeria, ADM is being implemented in Anambra State (one of the 36 states) by the Anambra State Health Insurance Agency (ASHIA)—a body responsible for managing and implementing the state health insurance scheme in the context of NHIS decentralization, as one of the efforts to scale-up the scheme through high-level political commitment, appropriate legislation, enabling policies and strategic plans for improved efficiency in health financing and delivery to achieve equity in healthcare services with assured financial risk protection toward the achievement of UHC (15). The model seeks to find a solution to the poor coverage of the informal sector within the Anambra state health insurance scheme (ASHIS). The implementation of the model commenced in early 2019 when a new administration took over the management of the ASHIA 2 year after its official launch.

Since the implementation of the ADM of health insurance, there is limited empirical evidence on the effectiveness of deploying the innovative model for improving coverage of the informal sector and resources within the scheme. Hence, an in-depth understanding of how ADM worked (is working), and the outcomes of its implementation is essential. Such evidence would be useful for government, policy/decision-makers, and health insurance program designers and implementers to inform policy and practice with respect to health insurance in the Nigerian states and other low- and middle-income countries (LMICs) with similar settings. This study aims to explore the influence of ADM on health insurance coverage and resources among rural dwellers and the informal sector in Anambra State, Nigeria. It also highlights contextual factors facilitating or constraining the implementation of such an innovative healthcare financing model.

## Overview of Anambra State Social Health Insurance Scheme

The Anambra State Health Insurance Scheme (ASHIS) was established by law in 2016, with the mandate to provide access to quality, affordable and efficient health services for every resident in the State (16). The scheme was officially launched by the state government in September 2018 to improve both physical and financial access to quality healthcare services for all residents toward achieving UHC by the year 2030 (17).

The membership of the ASHIS includes the vulnerable persons registered and resident in Anambra state; employees of the public and organized private sector (OPS) employing five (5) or more persons; and employees in the informal sector (17). The ASHIS was designed to be financed through premiums (social security, payroll taxes, and private contributions); government subsidy (general, earmarked taxes, and non-tax revenues), and other sources (donations, donor funds, etc.) (17). The premium to be contributed is a rate determined by an Actuary. The ASHIA operational guideline stipulates the contributions as follows: (1) Equity fund established for the vulnerable persons; (2) Earnings-related for the public (State & LGA) and OPS employees. The employer pays 10% of the basic salary while the employee contributes 5% of the basic salary. For employees of the organized private sector, the employer may decide to pay the entire contribution for the employees, and (3) Prescribed contributions as approved by the scheme's Council from time to time following an actuarial analysis for the informal sector program. The contribution of an annual premium of twelve thousand Naira per person is currently operational for the informal sector. There is a co-payment of only 10% of the cost of medications prescribed to an enrollee whether as outpatient or inpatient, made to the healthcare provider at the point of care (16, 17).

The scheme covers a basic package of services that is essential for Anambra residents including health promotion, disease prevention, curative, and rehabilitative health care services provided at the primary and secondary levels of care (16, 17). The law provides for the Anambra State Health Insurance Fund (ASHIF) as a single pool for the scheme funds. All contributions for the scheme are pooled into a dedicated account in an accredited bank for use. ASHIA pays the healthcare providers (HCPs) by capitation for primary care and Fee-for-service (FFS) for secondary and tertiary care. Capitation is paid in advance for a defined population for an agreed amount monthly to the HCPs while FFS is paid when claims have been submitted and processed by ASHIA (16, 17).

## Methods

### Study Area

This study was undertaken in Anambra State, southeast Nigeria. Anambra state has an estimated population of 4.5 million with an annual growth rate of 2.8% in 2018 (18). Structurally, the state health system is organized into three tiers; primary, secondary, and tertiary levels of healthcare. The State Ministry of Health (SMOH) co-ordinates primary and secondary healthcare services which are provided by both private and public health facilities. The major financing mechanism in the state before the launch of ASHIA is majorly budgetary allocations and OOPE for healthcare (15, 19).

### Study Design

We adopted a mixed-methods case-study design to enable in-depth exploration of how the ADM model was/is being implemented, its effectiveness in improving coverage of the informal sector and resources generation within the ASHIS as well as the barriers and enablers of its implementation. A case study design is deemed appropriate because the study is seeking to explore implementation experiences on “what,” “how,” and “why” outcomes of an event in a context-specific setting over a period of 3–6 months (20, 21). Mixed methods (qualitative data collection and secondary quantitative analysis of enrollment data) were used to allow for adequate triangulation and a robust description of findings. In Nigeria, ADM is a fairly new initiative and was initiated in Anambra state as an innovative intervention/strategy for expanding coverage of health insurance to the informal sector.

Qualitative data [in-depth interviews (IDIs) and document reviews] and quantitative methods (enrollment data from the ASHIA enrollment database) were collected to cover pre and during ADM events. In-depth interviews of key stakeholders on both the health insurance managers and adoptees and the document review were conducted to gain a deep understanding of how it was initiated or conceptualized, the process of its implementation, and the outcome of the ADM as well as the stakeholders' experiences in implementing ADM in the state. On the other hand, an analysis of secondary quantitative data was done to show the pattern of the informal sector coverage before and during the ADM intervention within the scheme.

### Study Population and Sampling

The study population was relevant stakeholders from the state and community levels who are involved in the planning and implementation of the ADM. For the IDI, the respondents were purposively selected based on their role in the organization and involvement in ADM planning and implementation (Table 1). At the state level, the respondents selected and interviewed include (i) Policy-maker: Executive Secretary of ASHIA; (ii) Departmental Heads (HODs) in the different departments of ASHIA: Accounts and Investment, Administration and Human Resource, Information, Communication, and Technology (ICT), Medical Services and Quality Control, Public Relation (PR), Planning Research and Statistics (PRS), and Business Development and Marketing (BDM) and; (iii) Media- health journalist. While at the community level some adoptees were purposively selected and interviewed based on their availability at the time of the study to explore their experience of the ADM intervention. The study did not employ specific standards in determining the sample size because the quality of the study is not influenced by the sample (22). Hence, the number of interview respondents was largely based on those with adequate knowledge of the subject matter and willingness to participate.

**Table 1:** The study in-depth interview respondents (*N* = 14).

**Organization/role**	**Total (*n*)**	**Sex (*n*)**	
		**Male**	**Female**
ASHIA staff	8	4	4
Philanthropist	4	4	–
Media officer	2	1	1
Total	14	9	5

### Data Collection

Data collection was done using tools developed by the researchers for the purpose of the study. IDIs with relevant stakeholders were conducted to explore the information on the origin of ADM was originated; processes and actors involved in the planning and implementation of ADM event/program; the outcome of the ADM in terms of an increased number of informal sector enrollees and resources; and contextual factors influencing ADM implementation. The interviews were conducted in pairs (an interviewer and a note-taker) by trained and experienced qualitative researchers based on convenient venues for the respondents. Prior to the interviews, participants were provided with an information sheet containing a brief description of the purpose and objective of the study, their rights as participants, and measures that will be taken by the study team to ensure confidentiality of the information provided. Verbal and written consent was obtained from each participant before the interview. We also sought and obtained permission to audio record interviews from each respondent. The audiotaped interviews were transcribed verbatim and handwritten notes taken during the interviews were built into the transcripts. The interviews were conducted in the English language at places convenient for the respondents including offices and houses and lasted for an average of 40 min per interview. A total of 14 respondents were interviewed (Table 1) between November 2021 and January 2022.

Rapid document reviews of relevant contextual literature were done following the York methodology, which includes five stages: identifying the research question; identifying relevant studies; selecting the publications for review; charting the data; and collating, summarizing, and reporting results (23). The documents were retrieved electronically from the organizational website and online publications relating to ASHIA and ADM between 2018 and December 2021. To ensure that relevant documents that capture the subject matter were not missed, the online searches were performed using various combinations of keywords. The keywords that were used to perform the electronic search include “informal sector,” “Adoption Model,” “enrollees,” “ASHIA,” “Anambra state,” and “social health insurance”. All retrieved documents (media articles) were included except duplicate publications, articles not related to Anambra State, and those whose themes were not pertinent to the scheme. The document search was performed by two reviewers (core review team) in December 2021. Data were extracted from relevant documents using an excel-based template developed by the core researchers that captured specific themes and questions as outlined in the data analysis section below. Retrieved sources were critically read to identify and document significant findings pertaining to ADM. Relevant information from each document was carefully summarized/paraphrased and entered into the excel file. Data extraction was also performed by the same reviewers who retrieved the documents. A total of 11 online documents were reviewed and included in the study.

For the quantitative data collection, the study undertook a secondary data extraction of enrollee information from the ASHIA enrollment database using a Microsoft Excel-based template developed by the researchers. The data extraction was done by the same team that did a rapid document review. The information collected was on the number of informal sector enrollees and the number of informal sector enrollees who registered through the ADM from January to December of the year 2018 and 2021.

### Data Analysis

Qualitative data were analyzed using the thematic framework approach as described by Braun and Clarke (24) using the inductive coding approach. All transcripts were anonymized with pseudonyms and were manually analyzed. The analysis of transcripts followed a rigorous process that started with familiarization of the transcripts to identify themes and codes that were based on the research objectives topic guide questions and recurrent themes; testing of the initial coding scheme; revision of the coding scheme and application to the rest of the transcripts. The coding of data was carried out by the authors and inconsistencies were resolved by consensus. The final codes that were used in the analysis were grouped into five broad themes: (1) conceptualization of ADM; (2) strategies/processes involved in planning and implementing ADM; (3) actors and their roles; (4) outcome of the ADM and; (5) contextual factors influencing ADM implementation.

For the quantitative data, a secondary analysis of the enrollee's information from the ASHIA enrollment database was analyzed using the summation, percentage, and chart functions of Microsoft Excel. ADM outcome was measured as counts per enrollment across all the HCPs: (i) a total number of informal sector enrollees per month per year; (ii) a total number of informal sector enrollees who registered through the ADM per month per year; (iii) the difference in the total number of informal enrollee's minus—the total number of enrollees that registered through the ADM and; (iv) the percentage difference between the total number of informal enrollees and a total number of enrollees that registered through the ADM. These were analyzed and represented in percentages, simple line graphs, and bar charts.

## Results

We present the result according to five broad themes deduced from the analysis of data as described above.

## Conceptualization of ADM for Health insurance

All the respondents stated that the ADM of health insurance originates from their current administration of ASHIA in November 2018. However, implementation commenced on February 2019. According to a respondent, “*ADM is a model designed by the agency under the leadership of Dr. AA, in the month of February 2019 to help the vulnerable people, that is, the poor in our society to have health insurance* (IDI, 02).

In the word of the Manager of ASHIA, *using the data of the NHIS which was about less than 1% enrollment of the informal population after over 15 years, we realized that if the people had money yet they were not signing up for health insurance, there was a mismatch between the ability-to-pay* (ATP) *and the willingness-to-pay* (WTP). *We asked ourselves when people who find it difficult to pay for healthcare are sick, who do they run to? they run to people who have the means to pay for them and these people who have the means may not always be willing to pay because they may not at that time be well able to pay when their dependents are sick so we said let us frame a structure of getting people to pay ahead for those who depend on them for settling healthcare bills. So, we tried to see how we can convince philanthropists to see how they can bring people on board the insurance scheme, that was the whole concept (IDI, 01)*.

The concept of ADM was reported to emanate from the fact that (i) the social milieu of Anambra state is such that there exists healthy rivalry among Anambra indigenes in philanthropy toward community development especially when rewarded. Findings from the rapid review have it that, “*ASHIA recognized that philanthropy abounds in some communities, and most times acts of philanthropy extend beyond the home of people, the Agency then utilized the opportunity to get wealthy people to pay an insurance premium for the less privileged”* (DRR, 01); (ii) high ATP among the citizens of the state and; (iii*)* quest to achieve UHC through increased coverage of health insurance in the state to 30% by 2022, then 10% yearly until the state achieves 90% coverage by 2030 (DRR,02).

Some respondents stated that the implementation of ADM also leverages the window of opportunity (election campaign period) to get politicians to pay premiums for people. An ASHIA staff said, when *ADM took off in the state, in 2019 when most of the politicians were contesting for different posts- the senate, house of representative or assembly etc was when we registered a lot of people into the scheme to get people to vote for them (*IDI, 08).

## Strategies/Processes Used in the Implementation of the ADM

**Figure 1:**
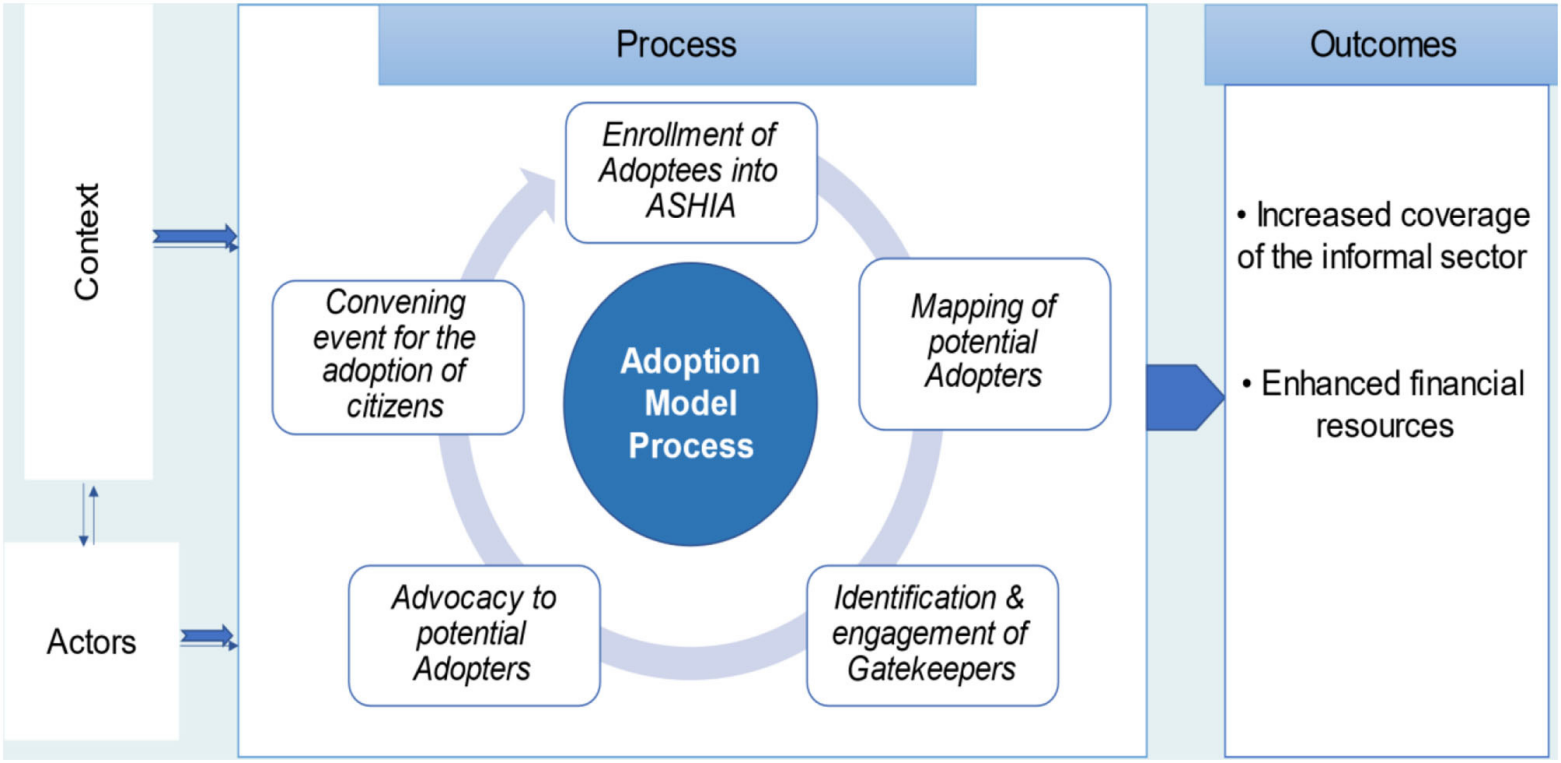
The adoption model of social health insurance scheme in Anambra State, Nigeria.

**Figure 2:**
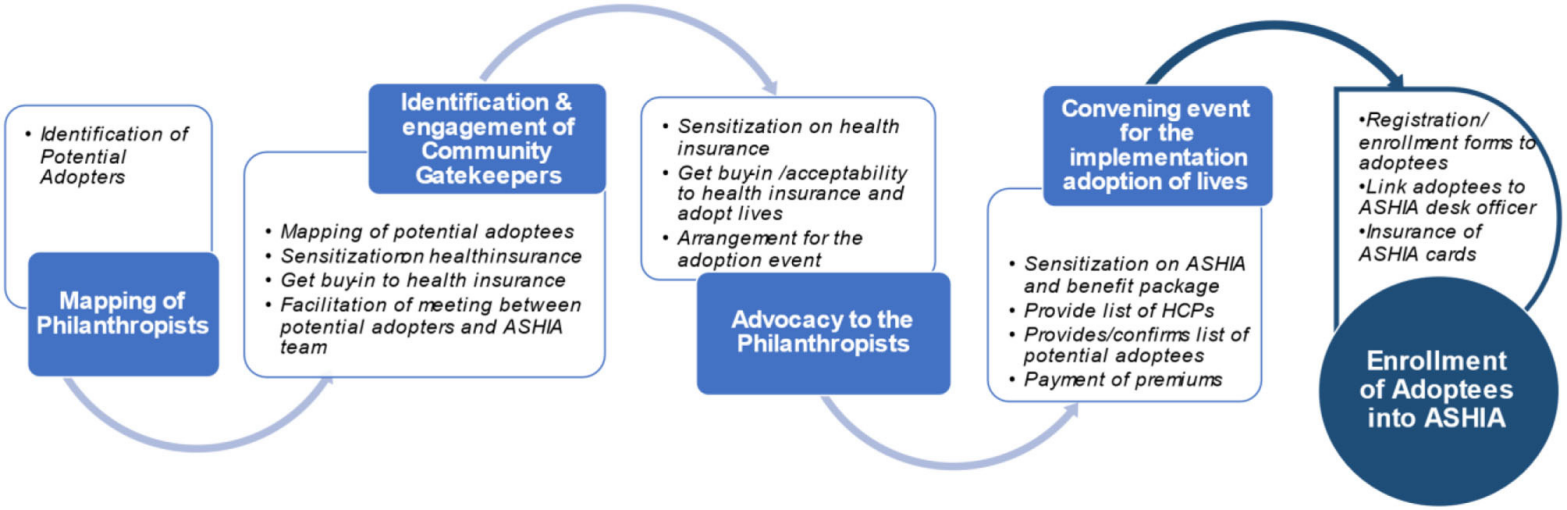
Summary of the chronological description of process of adoption model of health insurance.

According to the respondents, the process of implementation of the ADM for health insurance involves different phases that occur in five main chronological steps which include mapping of philanthropists, identification of gatekeepers, advocacy visit, convening events/meetings, and enrollment of adoptees. These steps are explained in detail below. The overall illustrative representation of the ADM is shown in Figure 1 while the implementation steps are summarized in Figure 2.

Step 1: Mapping of philanthropists in communities. The first step involved in the implementation of ADM begins with the mapping of philanthropists whereby the individuals that can potentially pay health insurance premiums for others are identified in different communities.

Step 2: Identification and engagement of community gatekeepers. ADM utilizes community gatekeepers and people of influence to facilitate or reach out to philanthropical indigenes of Anambra state to redirect their acts of charity toward payment of premiums for the state health insurance scheme for vulnerable or poor members of their communities. The gatekeepers include traditional rulers/*Igwes*, church leaders/ members of the clergy, President Generals (PGs) of Town Unions, opinion leaders, leaders of men and women groups in the communities, President of groups like the *Iyom* Association. The gatekeepers are first advocated and then engage to assist in the sensitization of the community members and philanthropists on the state health insurance scheme. Once their buy-in is secured, mapping of vulnerable persons in the community is done with their help.

“*After identifying those individuals, we go to their various communities, then identify community gatekeepers that can grant us access to reaching those people that we have identified”* (IDI, 03).

“*We approach them; educate them on health insurance and the need for individuals to have health insurance policy. We will then work with them to look at their communities to identify individuals who can pay for less privileged persons into the health insurance scheme”* (IDI, 05).

“*If you come to the community, you meet the Igwe to help get the philanthropists in the community, the affluent people, even government people who are rich. once we are able to get them, we work one on one and meet them and discuss with them about the ADM, how it would help them in their community and even the political people, we convince them that they can even use ADM to do the campaign and get electorates to vote them in because the more people you get in terms of ADM, it would help them to have more vote.”* (IDI, 07)

Step 3: Advocacy visits to the philanthropists. In this phase, the identified philanthropists are then approached in collaboration with some community gatekeepers to (1) sensitize them on the state health insurance scheme as a means of reducing catastrophic health expenditure and improving healthcare as well as the health insurance modus operandi including benefit package and (2) solicit to them to adopt indigent community members into the scheme.

Respondents reported that most times, the advocacy visit is done more than once to achieve the desired goal or set objective. A respondent also noted that sometimes at this stage, media are involved in order to disseminate the ADM visit aimed at shaping the public's perception of health insurance in the state. In the event of the successful conviction of the philanthropists to adopt people into the scheme, an appointment will be scheduled for the adoption event in the community or any other venue that is convenient to the community/potential *adopter*.

Step 4: Convening event/meetings for the adoption of people into the scheme: The next phase of the ADM implementation is to convene a meeting of all the potential *adoptees* on an agreed date, time, and venue comprising all stakeholders to (1) sensitize them on the Anambra State health insurance scheme: the benefits package; process and requirements for registration/enrollment and; (2) provide a list of healthcare providers in their locality where they can access healthcare, ASHIA desk officer, and helplines. After the ASHIA presentation or enlightenment about health insurance, the *adopters* will then make a pronouncement of the number of persons he/she wishes to pay premiums for. In most cases, some adopters pay through bank check (immediately or post-dated) while the others will immediately credit the ASHIA account through an online bank transfer. Below are some of the *adopter*'*s* statements during some of the ADM convenings according to findings from the rapid review:

*It is my pleasure to enroll over 200 indigent individuals into ASHIA. I enjoin you (the adoptees) to access health services at the designated hospital once you are sick in order to reduce the financial stress one goes through to get quality healthcare services* (DRR, 04).

A member in the State House of Assembly states at their Civic Center, “*I am enrolling these 150 persons into ASHIA and will pay the premium on their behalf, for them to enjoy all the health benefits packages offered by ASHIA. Please always pay attention to your health by visiting the ASHIA accredited hospitals where you are registered regularly for a check-up too”* (DRR 06).

Step 5: Registration/enrollment of *adoptees* into ASHIA. The last stage of the ADM process is the registration of the *adoptees* as well as linking them to an ASHIA desk officer allocated to that particular LGA/community. According to an ASHIA staff,

“*We give them* (adoptees) *our enrollment forms, they complete it and return same to ASHIA through individuals that we would now designate as link persons between ASHIA and the adopters. Those individuals now work with the adoptees to ensure complete the enrollment process. We give them their ASHIA enrollment cards and counsel them on their rights and privileges within the scheme. These adoptees then go to the hospitals and access care after their waiting period is over* (IDI, 01).

## Actors and Their Roles in the Implementation of ADM

The respondents stated that the major identified actors were those who are directly involved in both the planning and implementation stages of the ADM of health insurance. The actors include the Executive Governor of the State, the Executive Secretary and Heads of Departments of ASHIA, community gatekeepers as described above, and the media. According to them, the diverse actor's arrangement aims at achieving a common agenda of interest. Most times, the team is not merely a combination of all the actors but depends on the community or philanthropists involved with a shared mission of engaging potential adopters or philanthropists and identifying poor/vulnerable citizens in their community toward successful adoption of individuals into the state health insurance scheme. In the words of a respondent,

“*The people involved are the executive secretary of ASHIA, the head of the department of the marketing and business development of ASHIA with all the members of the team. Then the critical gatekeepers we have identified at the community level: traditional rulers, church leaders, president generals of town unions, opinion leaders, women leaders, and leaders of men and women groups in the communities. Also, the staff that go to do the enrollment in the different communities. Sometimes, we equally talk about ad-hoc staff that we recruit when we have a large number to enroll like the time when we had people paying for over a thousand lives* (people*), we needed some ad-hoc staff to come in and equally help to ensure the process was completed very seamlessly and efficiently.”* (IDI, 05)

The overall goal of diverse actors is to (i) enable increased identification and access to the identified philanthropists; (ii) increase awareness for health insurance through information sharing and; (iii) ensure accountability. These actors' engagement was reported to be instrumental to the success of ADM implementation. The various actors and their roles in the successful implementation of the ADM are summarized in Table 2.

**Table 2:** Key actors involved in the ADM of health insurance and their roles.

**Actors**	**Roles and responsibilities**
Governor	• Markets and convince potential philanthropists to adopt lives • Flags off some ADM enrollment exercise
Executive secretary, ASHIA	• Supports awareness creation/sensitization of the state health insurance • Leads in the mapping of potential adopters/philanthropists • Gets approval from the Governor and solicits assistance in convincing some identified potential philanthropists • Contacts and markets the scheme to the philanthropists • Leads in advocacy and engagement with identified philanthropists and community gatekeepers for the ADM • Provides technical support to staff during the planning and implementation of ADM • Approves date for the ADM events • Supports organizing of ADM events • Participates in ADM flag-off events • Supervises the enrollment/registration of the adoptees • Receives feedback from the community gatekeepers
HOD, marketing, and business development, ASHIA	• Leads in marketing the scheme to the informal sector • Leads in awareness creation/sensitization of the scheme • Enrolls/registers adoptees • Follow-up on registered adoptees
Other ASHIA staff	• Enrolls/register adoptees
Community gatekeeper	• Lead ASHIA staff/Executive secretary to the identified philanthropists in the community • Support awareness creation about health insurance to the philanthropists • Identify/map out poor and vulnerable members of the citizen for enrollment into the scheme. • Assist in organizing the ADM meeting/event • Feedback report on the progress or challenges in accessing care at the chosen HCP(s)
ASHIA *ad hoc* staff	• Supports enrollment/registration of adoptees to ensure a well-organized process
Media	• Enlighten public of the health insurance • Dissemination of ADM events

## Outcomes

### Increased Coverage of Informal Sector Enrollment

Using ADM as a strategy for improving coverage/enrollment of the informal sector into the state health insurance scheme was reported to have led to an increased number of enrollees in the pool, as it created awareness of the scheme and motivated philanthropists to adopt people into the scheme. According to a top manager of ASHIA, *before we started the adoption model in February 2019, we had*<*200 persons who enrolled in our scheme from the informal sector. The moment we started the ADM, as of this moment, we have an adoption model accounting for over 80% of our informal sector enrollees which currently stands at over 65,000 individuals because we have all the records of how these adoptions happened (IDI, 01)*.

In the words of an ASHIA respondent, *For the past 3 years since we started ADM, it has been very successful because it has increased the number of non-government enrollees in ASHIA's database…. Very successful in the state, and many people are coming down from different States to learn from us and see how we are going about this model*. (IDI,08).

Another respondent alluded to the fact that there was a marked increase in the number of enrollees from the informal sector, she said, *so far, the outcome of the ADM is quite encouraging. It has really skyrocketed the number of informal enrollees on our platform. for instance, there are some philanthropists that I know that enrolled up to 1,000 lives, some 500 lives depending on financial capacities and there are some schools that already have more than 1,200 lives, etc.… the arrangement has been made in such a way that it is included in their school fees so automatically their premium would be coming to the agency every session. So that adoption model is a very nice one …It is a really wonderful idea* (IDI, 03).

The majority of the respondents stated that not only that there is a high willingness to adopt lives (WTDL) among these philanthropists, that there is also a high renewal rate of the annual premium for the adoptees. However, in situations where the adopters do not or are not able to renew the premiums, adoptees do so themselves because of the increased awareness or felt benefits of health insurance in improving their health. A respondent said,

*The retention rate is quite high because over 80% of the adoptees have their premiums renewed, either by the philanthropists paying for them or some even pay by themselves the following year and keep paying their premium and this is because they now understand the need for health insurance and this has greatly improved the health insurance culture in Anambra state* (IDI, 05).

**Figure 3:**
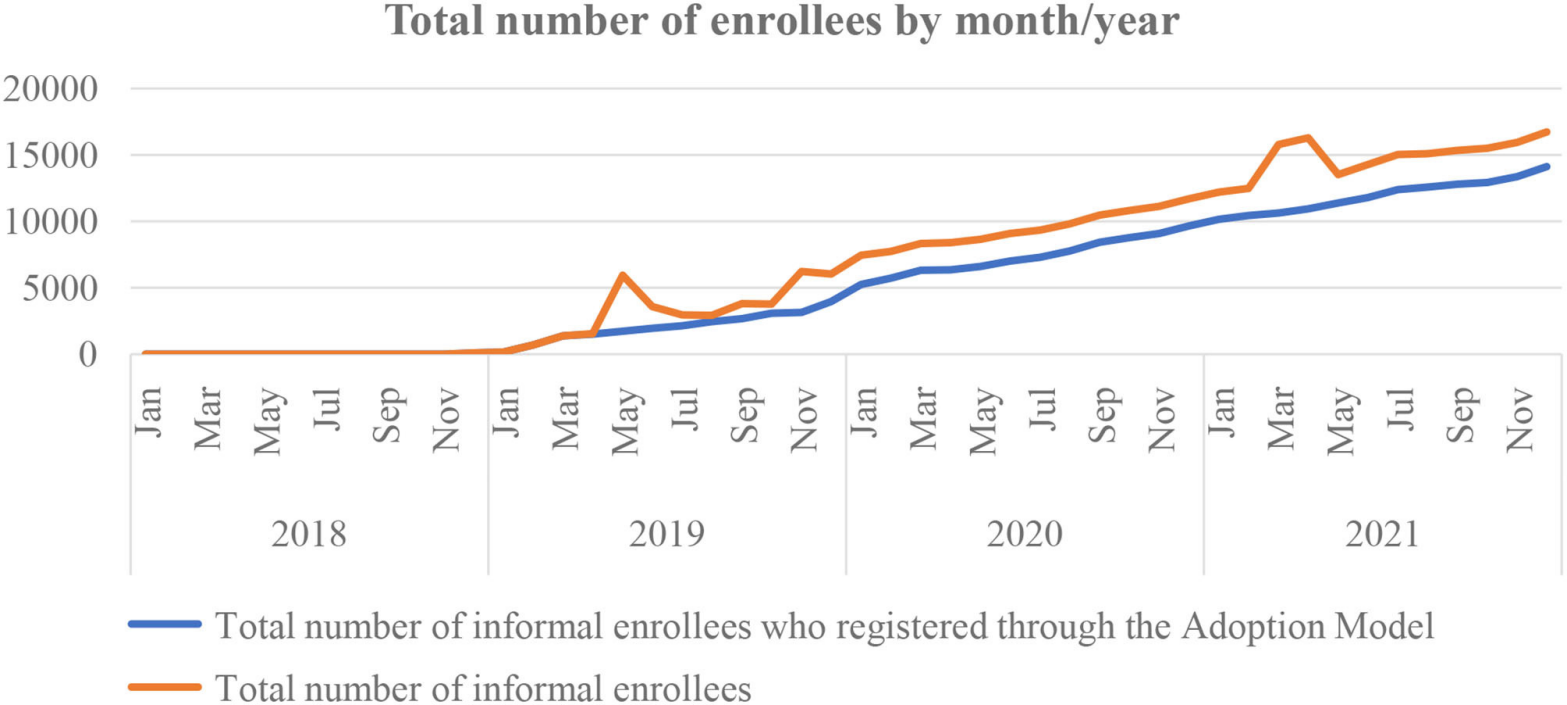
Trend of health insurance coverage before and during the adoption model intervention among informal sector enrollees between the year 2018 and 2021 in Anambra State Health Insurance Scheme.

**Figure 4:**
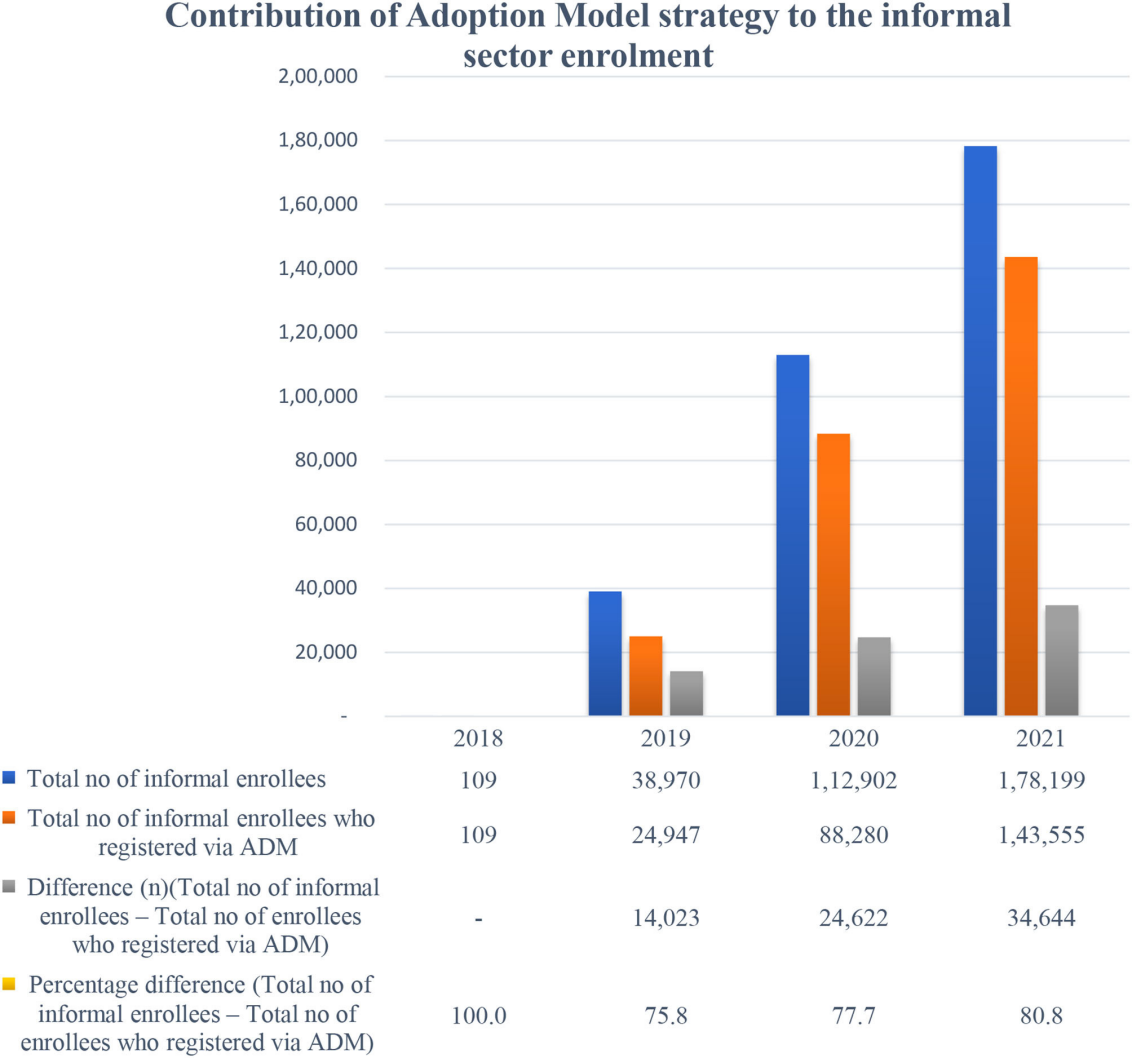
Contribution of adoption model intervention to the informal sector enrollment in Anambra state health insurance scheme between the year 2018 and 2021.

The findings from quantitative data showed a remarkable trend of rising enrollment in the informal sector from the last quarter of the year 2019 through 2020 in the state health insurance (Figure 3). The continuous flight mode increase in the enrollment of the informal sector was observed shortly after the commencement of the adoption model for health insurance in February 2019. Further secondary analysis of informal enrollees' data shows that over a period of 4 years (2018–2021) of ADM implementation, it has contributed an average of 79.3% of the informal enrollees to the scheme; 100% in 2018, 75.8% in 2019, 77.7% in 2020, and 80.8% in 2021 (Figure 4).

### Increased Financial Resources of the Scheme

In addition, the ADM strategy has led to an increment in fund generation. For instance, in January 2022, the agency received its largest contribution since the ADM began. According to the rapid review report, a philanthropist and a Chief Executive Officer of the “DD” Group, who also runs a Foundation, “*enrolled 1,500 members of his hometown on the scheme,”* which brings a total of N18,750,000 (USD $37,000) into the funding pool (DRR, 01). This implies that over 65,000 enrollees from the informal sector in the scheme have generated ~N780,000,000 (USD $1,400,000) in the funding pool.

## Contextual Factors Influencing the Implementation of ADM

### Enablers

Some enablers to the implementation of the ADM of health insurance among the informal sector were noted as follows: (i). High level of ATP; (ii) Availability of public-spirited philanthropists in the state; (iii) Legal backing- the Anambra State Health Insurance Law enables the Agency to operate optimally; (iv) High political commitment and support, “*The zeal and commitment of a supportive Governor helps, and His Excellency has shown incredible support for what ASHIA is doing. He does not only support what we are doing, but he also adopts lives. So, without such support, we won*'*t succeed. Even our politicians representing us at both Federal and State Houses of Assembly are adopting lives and paying annual premiums on daily bases”* (IDI, 01); (v) Organizational factors including, conducive/enabling work environment (incentives, supervision, office space, etc.), and experienced and trained marketers in the agency; (vi) Massive awareness creation of the scheme through the use of mass or social media, public events including churches, roadshows, and radio jingles; (vii) Good teamwork among ASHIA staff and; (viii) Testimonial from enrolles on quality of services provided by the ASHIA healthcare providers.

### Barriers

Respondents opined that a major barrier to implementing ADM is rejection or refusal by some identified philanthropists due to a lack of trust in government. These philanthropists were skeptical about government programs usually marred by corrupt or sharp practices. A respondent said *you know the government thing! they will carry the money before you know it and disappear and they say the thing is closed and nothing will happen to them* (IDI, 02).

The second major challenge of the ADM reported by some respondents is health systems barriers. First, the lack of standard/proximity of hospitals in some communities makes access to healthcare difficult, hence the reluctance of some philanthropists in the community to adopt people. For instance, an ASHIA respondent narrated his experience thus,

*Okay, there was a time I went to one of the Igwe's in Ayamelum local government, someone actually wanted to sensitize the Igwe so the person reached out to me and I had to follow her to the place. At the end of the sensitization, the Igwe was kind of reluctant because according to him there is no good hospital his people would be attending because for him if he's going to pay for his people the people must be attending hospitals in his community, that he cannot pay for his people and his people would be stressing to go to neighboring communities to access care that he doesn't buy into that. So, it became a big challenge because they don't have government hospitals in their place* (IDI, 07).

In addition, another respondent said, *in some of the communities, we do not have hospitals that meet the accreditation criteria for ASHIA to be a provider in our scheme. So, for some of those communities, the fact that there are no readily available health care providers to take on our enrollees that can render primary, secondary and tertiary services like those in the localities would want to have access to, discourages some of the individuals. So those are some of the challenges we've had (IDI, 01)*.

Second, poor attitude of health workers on the premises of the health care providers for those who have gone to seek care, very few though, but those health care providers who have not treated the enrollees properly; some of these enrollees discourage other individuals who are yet to be registered aboard the scheme not to do so.

Another challenge the model experiences is the ever-shifting and unpredictable priorities of politics. In the words of a respondent “*When you market health insurance and ADM to politicians, their priorities may differ from yours, and making you to constantly pay advocacy to them on the benefits of health insurance to appeal to their conscience; sure, you face lots of disappointments. M*ost times their politician's mindset is that *health is not usually instrumental to winning elections until you let them know, that adopting lives can win people's votes for them. Interestingly, out of the 11 politicians who eventually adopted lives, nine of them won their elections. …. you see*.” (DDR,10)

Other barriers identified include i) Socio-cultural/religious barriers. The sociocultural and religious characteristics of the people were reported to preclude them from wanting to have a health insurance policy. To buttress this barrier, a respondent stated,

*In some cases when you go to certain individuals to market health insurance to them and, outrightly, they say "are you praying from me to be sick?” ….that having health insurance means that I have accepted that I would be sick. They believe that paying for health care in advance attracts sickness or disease to their families So those belief systems that have been engrained in those citizenries have hampered a lot of the progress that we hoped to have made through ADM (IDI, 01)*.

(ii) Reform spoilers such as unaccredited private hospitals discourage people from enrolling in the scheme so that they will still be patronizing them. In the words of a respondent,

*Most of them prefer the high charges that they bill people when they come through the what I call “cash and carry” (profiteering mindset) health system that operates, unlike the health insurance scheme that would ensure the strategic purchasing of healthcare services. In this case, people would prefer to render services but bill people exorbitantly, for instance, a cesarean section in our scheme costs N70,000 but in this same state, there are providers who charge N500,000 for one cesarean section. so you realize that such individuals may not want members of the community to key into strategic purchasing that ASHIA offered people rather they would want them to remain with the cash and carry health system, collecting more money from them at once not knowing clearly that health insurance is a sure sustainable way to keep their facilities afloat but for them that strategic purchasing which is critical about health insurance becomes a limitation so they discourage some members of the society (IDI, 01)*.

However, the majority of the respondents stated that one major strategy adopted by the ASHIA to mitigate/prevent the challenges affecting ADM is continuous sensitization and advocacy for health insurance as well as its benefits through various channels including radio jingles, social media, newspaper publications, television broadcasts, church gatherings, community meetings, etc.

## Discussion

The study demonstrated how the concept of ADM was utilized as an innovative strategy to enhance enrollment of the informal sector for improved coverage of the informal sector and enhanced financial resources within the SSHIS in Anambra state, Nigeria. There are indications that the strategy has achieved its intended objective of targeting a pool of high-income indigenes of the state to purchase annual health insurance subscriptions for the low-income population to improve access to quality healthcare toward achieving UHC.

The study revealed that implementing ADM contributed to increased coverage of the informal sector in the sub-national health insurance scheme. This could be attributed to the high level and continuous sensitization and advocacy embedded in the ADM intervention. Advocacy/sensitization has been shown to be an important strategy to increase public awareness of health programs and it is usually part of a process targeting to expand community engagement (25). The implication is that ADM was a good strategy for improving both awareness and increasing enrollment of the informal sector in the health insurance. In Nigeria, low participation and retention are challenges affecting health insurance operation and sustainability (26). However, with the ADM strategy, is likely that a large pool of informal sector enrollees would be retained in the scheme provided their annual premiums are renewed. Such successful experiences with similar implementation features had been reported elsewhere (14, 27, 28).

Adoption model strategy offered a great opportunity for payment of health insurance premiums, improving revenue generation, and facilitating financial sustainability for the SSHIS. The overwhelming success of the innovation suggests a high level of acceptability and WTALs among public-spirited philanthropists to support the health goal of improving access to healthcare among the less privileged or vulnerable indigenes. Our finding is in collaboration with the report that philanthropic investments/financial contributions enable the pooling of their resources toward a just cause provided the pooled funds are disturbed in a coordinated and efficient way (29). Our findings suggest that philanthropic ADM intervention made a valuable contribution to supporting or strengthening the state health insurance resource mobilization and thus improving financial sustainability within the scheme. In the context of UHC, ADM undoubtedly removed financial barriers by reducing high out-of-pocket expenditures (OOPE) and addressing the huge health inequities among the beneficiaries which are the major reasons for poor access to healthcare services, especially among the rural geographical areas in developing countries including Nigeria (30, 31).

The key elements of the ADM are the broad context within which the strategy is conceived and implemented, the interaction of the actors involved, and the implementation process. The context assumes that innovation development and implementation occur within a policy subsystem. Hence, understanding and leveraging the political and economic dynamics of policy is fundamental to achieving desired policy change (21).

The process of implementing ADM instilled the desired behavior/acceptability of health insurance among various stakeholders involved at different stages of executing this innovative strategy. Also, the roles of various stakeholders including community gatekeepers and influencers were prominent in the effectiveness of the ADM process. Most importantly, the embedded sensitization on health insurance at all most every stage of the ADM design created or enabled improved public enlightenment about the scheme and facilitated the process of stakeholders' buy-in to the scheme including the adopters. A report had shown that lack of awareness contributes to the current low uptake of health insurance in Nigeria (26). However, the poor awareness of health insurance particularly among the informal sector and rural areas was addressed by the high sensitization of health insurance and its benefits and had been shown to be valuable tools in behavioral change. More so, the inclusive stakeholders' engagement and collaborative nature of designing and implementing the ADM contributed greatly to the success of the strategy. This implies that ADM stimulated both the adoptees and adoptees into enrollment in the scheme. Collaboration and proper coordination in health programs are required for a successful program implementation because instilled the spirit of ownership and learning. This finding corroborates other reports that collaboration benefits stakeholders whilst influencing policy and practice of a health intervention (32, 33). This implies that for the sustainability of ADM for health insurance coverage and resource mobilization, a stronger collaborative effort that will ensure relevant stakeholders' inclusiveness should always be incorporated from conceptualization to implementation stages for acceptability and buy-in to the scheme. This is because these actors act as catalysts of policy trajectory, as they provide a stimulus to change situations outside the control of a health system. Our finding has important public health implications in regions where uptake of health insurance is low.

One of the major drivers of success is the strong political will and commitment demonstrated by the Anambra state governor and other policy/decision-makers who allowed and supported the scheme to implement ADM and other successful programs without bureaucratic bottlenecks, limited autonomy, and political interferences (34) experienced in most government health programs. Our finding is in line with a report that strong administrative and political support demonstrated through technical support was instrumental to the successful implementation of a community-based health insurance scheme for the informal sector in Rwanda (35). Other enablers of the implementation of ADM include numerous public-spirited philanthropists, legal institutionalization of health insurance in the state, good teamwork among ASHIA staff, enabling work environment (incentives, supervision, office space, etc.), and experienced and trained marketers in the agency also facilitated the success story of the ADM. This implies that adopting ADM as a strategy for strengthening their health insurance scheme should also ensure that these enable identified are in place before implementing such innovation.

However, the implementation of this innovative strategy/intervention has been constrained by some challenges. Distrust for government health programs and the health system had shown to continually pose major challenges in the acceptability and uptake of health inventions in Nigeria (36). Likewise, inimical cultural and religious beliefs about health insurance and health systems barriers to accessing health care ranging from the poor attitude of health workers, substandard health facilities and equipment, etc. are the major barriers that negatively affected the ADM implementation. Our findings collaborate with the previous one that reported reasons for poor enrollment of health insurance including normative thoughts or perceptions regarding wrong or superstitious beliefs about health insurance attracting illness, low level of trust in government health programs and policies (9, 10, 26) and religious and cultural beliefs (26, 37).

Our study shows that in the short lifecycle of ASHIA existence, the scheme has experienced significant progress in enrollment of the informal sector through the ADM strategy toward achieving its objectives for UHC. Thus, the model remains a distinctive feature of ASHIA which has enhanced financial sustainability and better operations through improved coverage of the informal sector for improved access to quality healthcare delivery to poor beneficiaries. However, the big question is then, how sustainable is such innovation particularly in the context when it loses its support from state government probably due to a change of government or management of ASHIA? This is because of previous experience from financial reform implemented in the past was abruptly terminated due to change in government (38).

A major strength of this study is the using mixed-method which contributed largely to the robustness and richness of the study because it enabled a wholistic exploration/assessment of the ADM intervention/study objectives. For instance, the secondary quantitative data analysis revealed trends in the informal sector enrollment. Employing both qualitative and quantitative methods increased the study's trustworthiness, conformability, and transferability.

However, one limitation of the study is that we did not elicit information from the beneficiaries (adoptees) on the process of the adoption which would have been more enriched if they were interviewed to better understand their experiences with the ADM implementation. Hence, could be an area for further research.

## Conclusion

The study provides context-specific evidence that demonstrates how the implementation of innovative ADM enhanced financial resources and sustainability and coverage of informal sector enrollment to the SSHIS. Hence, the study offers new evidence that can inform policy/decision-making for planning and implementation of the intervention(s) aimed toward improving coverage and strengthening health insurance schemes in LMIC with similar settings. It is therefore recommended that the strategy should be adopted in settings where philanthropy abounds for increasing both physical and financial access to quality healthcare delivery, particularly among poor beneficiaries toward achieving UHC in Nigeria as well as other developing countries.

## Data Availability Statement

The raw data supporting the conclusions of this article will be made available by the authors, without undue reservation.

## Ethics Statement

The studies involving human participants were reviewed and approved by Health Research and Ethics Committee of the State Ministry of Health, Awka, Anambra State. The Ethics Committee waived the requirement of written informed consent for participation.

## Author Contributions

SBO and URE conceptualized the study and drafted the manuscript. SBO participated in data collection while URE analyzed the data. All authors reviewed and approved the final version of the manuscript before submission.

## Funding

The research leading to results included in this study was funded fully by the authors. All views expressed in this article are of the authors only and do not necessarily represent views of ASHIA.

## Conflict of Interest

The authors declare that the research was conducted in the absence of any commercial or financial relationships that could be construed as a potential conflict of interest. The reviewer PA declared a shared affiliation with the author URE to the handling editor at the time of the review.

## Publisher's Note

All claims expressed in this article are solely those of the authors and do not necessarily represent those of their affiliated organizations, or those of the publisher, the editors and the reviewers. Any product that may be evaluated in this article, or claim that may be made by its manufacturer, is not guaranteed or endorsed by the publisher.
